# Fit-For-All iPSC-Derived Cell Therapies and Their Evaluation in Humanized Mice With NK Cell Immunity

**DOI:** 10.3389/fimmu.2021.662360

**Published:** 2021-04-02

**Authors:** Charlotte Flahou, Tatsuya Morishima, Hitoshi Takizawa, Naoshi Sugimoto

**Affiliations:** ^1^ Department of Clinical Application, Center for iPS Cell Research and Application (CiRA), Kyoto University, Kyoto, Japan; ^2^ Laboratory of Stem Cell Stress, International Research Center for Medical Sciences (IRCMS), Kumamoto University, Kumamoto, Japan; ^3^ Laboratory of Hematopoietic Stem Cell Engineering, International Research Center for Medical Sciences (IRCMS), Kumamoto University, Kumamoto, Japan

**Keywords:** humanized mice, human induced pluripotent stem cells, regenerative medicine, natural killer cells, human leukocyte antigen****

## Abstract

Human induced pluripotent stem cells (iPSCs) can be limitlessly expanded and differentiated into almost all cell types. Moreover, they are amenable to gene manipulation and, because they are established from somatic cells, can be established from essentially any person. Based on these characteristics, iPSCs have been extensively studied as cell sources for tissue grafts, blood transfusions and cancer immunotherapies, and related clinical trials have started. From an immune-matching perspective, autologous iPSCs are perfectly compatible in principle, but also require a prolonged time for reaching the final products, have high cost, and person-to-person variation hindering their common use. Therefore, certified iPSCs with reduced immunogenicity are expected to become off-the-shelf sources, such as those made from human leukocyte antigen (HLA)-homozygous individuals or genetically modified for HLA depletion. Preclinical tests using immunodeficient mice reconstituted with a human immune system (HIS) serve as an important tool to assess the human alloresponse against iPSC-derived cells. Especially, HIS mice reconstituted with not only human T cells but also human natural killer (NK) cells are considered crucial. NK cells attack so-called “missing self” cells that do not express self HLA class I, which include HLA-homozygous cells that express only one allele type and HLA-depleted cells. However, conventional HIS mice lack enough reconstituted human NK cells for these tests. Several measures have been developed to overcome this issue including the administration of cytokines that enhance NK cell expansion, such as IL-2 and IL-15, the administration of vectors that express those cytokines, and genetic manipulation to express the cytokines or to enhance the reconstitution of human myeloid cells that express IL15R-alpha. Using such HIS mice with enhanced human NK cell reconstitution, alloresponses against HLA-homozygous and HLA-depleted cells have been studied. However, most studies used HLA-downregulated tumor cells as the target cells and tested *in vitro* after purifying human cells from HIS mice. In this review, we give an overview of the current state of iPSCs in cell therapies, strategies to lessen their immunogenic potential, and then expound on the development of HIS mice with reconstituted NK cells, followed by their utilization in evaluating future universal HLA-engineered iPSC-derived cells.

## Introduction

The ability to reprogram adult cells into induced pluripotent stem cells (iPSCs) has changed regenerative medicine. The original reprogramming to induce pluripotency in somatic cells was done by the ectopic co-expression of four transcriptions factors, c-MYC, OCT3/4, SOX2 and KLF4, or similar combinations (1–4). iPSCs (re-)acquire the ability to develop the three embryonic germ layers and propagate in culture indefinitely. Their cell fate can then be further directed to differentiate iPSCs into various types of differentiated human cells (5). In addition, iPSCs can be established from essentially anyone and genetically manipulated. iPSCs are therefore regarded as a suitable platform to develop regenerative cell therapies.

As iPSC-derived cells make their way to clinical applications, allogeneic cells as off-the-shelf products are being preferentially considered (6, 7). Thus, a critical challenge is making iPSCs immunologically tolerable by the receiving individual. To circumvent the most significant alloimmune barriers to allotransplantation, namely the mismatch of HLA, a number of strategies are being explored. iPSCs can be established from people with homozygous HLA haplotypes, which would increase the number of matching recipients to a single donor, and are available through iPSC stocks (8–10). In addition, engineering the HLA expression of the cells to be transplanted, specifically downregulating HLA, could also be immunologically tolerable for the recipient. However, even supposing that the T cell-based primary rejection mechanism is avoided by HLA downregulation, natural killer (NK) cells possess complementary recognition pathways which can promote graft rejection (11–13).

Accurately recapitulating NK cell rejection mechanisms *in vivo* is therefore crucial for successful HLA-homozygous and HLA-engineered iPSC-based transplantation therapies. To this end, notable efforts have allowed for mice with a humanized immune system (HIS) to acquire an increasingly comprehensive representation of human immune cell populations (14). From interventions aiming to entirely mute the mouse immune system to sophisticated genetic engineering to switch mouse immune factors to their human version, strategies have yielded mouse models that can recapitulate specific parts of human immunity. Notably, the reconstitution of the innate immune compartment, such as myeloid cells and NK cells, has recently gathered a fair amount of attention.

This review aims to provide the reader with an overview of the therapeutic potential of iPSCs with strategies to overcome HLA barriers in regenerative medicine, along with their evaluation in HIS mice sufficiently reconstituted with human NK cells.

## Current Status of the Clinical Application of iPSCs in Regenerative Medicine

Cell replacement therapy and regenerative medicine advances are excitingly multiplying as research explores their great proliferation potential and high versatility of iPSC application. Contrary to the relatively controversial embryonic stem cells (ESCs), iPSCs do not involve the handling of embryonic material and therefore provide a more acceptable platform to develop regenerative therapies. Nevertheless, norms and expectations surrounding the ethical implications of iPSCs have been accounted for since the early times of their development (15) and regularly discussed since then (16, 17). In addition to their minimal ethical hurdle, iPSCs – like ESCs – can be produced off-the shelf in clinically relevant quantities in bioreactors or culture dishes, which enables a stable supply and use for emergency situations, given the right logistics. They can also be differentiated into various types of adult human cells (5) as well as qualified for safety (pathogens and contamination) and consistency (genotyping, phenotype characterization). iPSCs are also relatively receptive to genetic engineering, which opens endless possibilities for clinical applications. Reports from pre-clinical and *in vitro* studies, reviews (6, 18, 19), as well as two clinical trials for age-related macular degeneration (AMD) (20, 21) and, more recently, mesenchymal stromal cells (MSCs) for graft versus host disease (GVHD) (22) have so far indicated that iPSC-based therapies are safe.

Although iPSCs are only a little over a decade old, as of December 2020, about twenty clinical trials have been publicly announced as either completed or ongoing, including one targeting COVID-19 using iPSC-derived MSCs (23) (**Table 1**). AMD was the first pathology to be given approval for human transplantation of iPSCs-derived cells (20, 21). To date degenerative eye disorders are of the most commonly targeted diseases in iPSC-based clinical trials (24–27), along with cardiac syndromes such as heart failure and myocardial ischemia (28–32), followed by neuropathies - Parkinson’s disease and spinal cord injury (33, 34) -, and thrombocytopenia from aplastic anemia (35). In contrast to the replacement of defective cells illustrated by those studies mentioned previously, iPSCs-based development of immunotherapies, namely iPSC-derived MSCs and iPSC-derived NK cells, have been conducted more proactively by the private sector for GVHD (36) and hematologic malignancies (37–40), respectively. Of note, within all the trial mentioned, three used autologous patient-derived iPSCs as the source of the cell for transplantation (two trials for AMD and the trial for thrombocytopenia from aplastic anemia), while the other trials transplanted cells differentiated from banked iPSCs.

**Table 1:** Registered clinical trials using iPSCs.

Affiliation	Disease target	Cell therapy	ID	Date	Cell source
**Help Therapeutics**	Heart failure	iPSC-CMs	NCT03763136	(estimated) May 1, 2019	Allogeneic
**University Medical Center Goettingen**	Heart failure	iPSC-CMs and stromal cells in hydrogel	NCT04396899	(estimated) December, 2020	Allogeneic
**Osaka University**	Myocardial ischemia	iPSC-CMs	NCT04696328	2-Dec-19	Allogeneic
**National Eye Institute**	AMD	iPSC-RPE on PLGA scaffold	NCT04339764	23-Sep-20	Autologous
**Cynata Therapeutics**	Steroid-resistant GVHD	iPSC-MSCs	NCT02923375	1-Mar-17	Allogeneic
**Fate Therapeutics**	Various cancers	iPSC-NK cells	NCT03841110	15-Feb-19	Allogeneic
**Fate Therapeutics**	AML, B-cell lymphoma	iPSC-NK cells	NCT04023071	4-Oct-19	Allogeneic
**Fate Therapeutics**	B-cell lymphoma or CLL	iPSC-NK cells	NCT04245722	19-Mar-20	Allogeneic
**Fate Therapeutics**	AML, multiple myeloma	iPSC-NK cells	NCT04614636	(estimated) November, 2020	Allogeneic
**Cynata Therapeutics**	COVID-19, ARDS	iPSC-MSCs	NCT04537351	24-Aug-20	Allogeneic
**Keio University School of Medicine**	Heart failure	iPSC-CMs	jRCTa032200189	9-Nov-20	Allogeneic
**Kobe City Eye Hospital**	Retinitis Pigmentosa	iPSC-RPE	jRCTa050200027	5-Oct-20	Allogeneic
**Kyoto University Hospital**	Thrombocytopenia	iPSC-platelets	jRCTa050190117	14-May-19	Autologous
**Keio University School of Medicine**	Spinal cord injury	iPSC-neural stem/progenitor cells	jRCTa031190228	1-Dec-20	Allogeneic
**Kyoto University Hospital**	Parkinson's disease	iPSC-dopaminergic progenitors	JMA-IIA00384	12-Sep-18	Allogeneic
**RIKEN**	AMD	iPSC-RPE	UMIN000011929	2-Oct-13	Autologous
**Kobe City Eye Hospital**	AMD	iPSC-RPE	UMIN000026003	6-Feb-17	Allogeneic (HLA-matched)

AMD, age-related macular degeneration; GVHD, graft versus host disease; AML, acute myeloid leukemia; CLL, chronic lymphocytic leukemia; ARDS, acute respiratory distress syndrome; CMs, cardiomyocytes; MSCs, mesenchymal stromal cells; RPE, Retinal Pigment Epithelium.

## Strategies to Circumvent Immune Rejection Against iPSC-Derived Grafts

**Figure 1:**
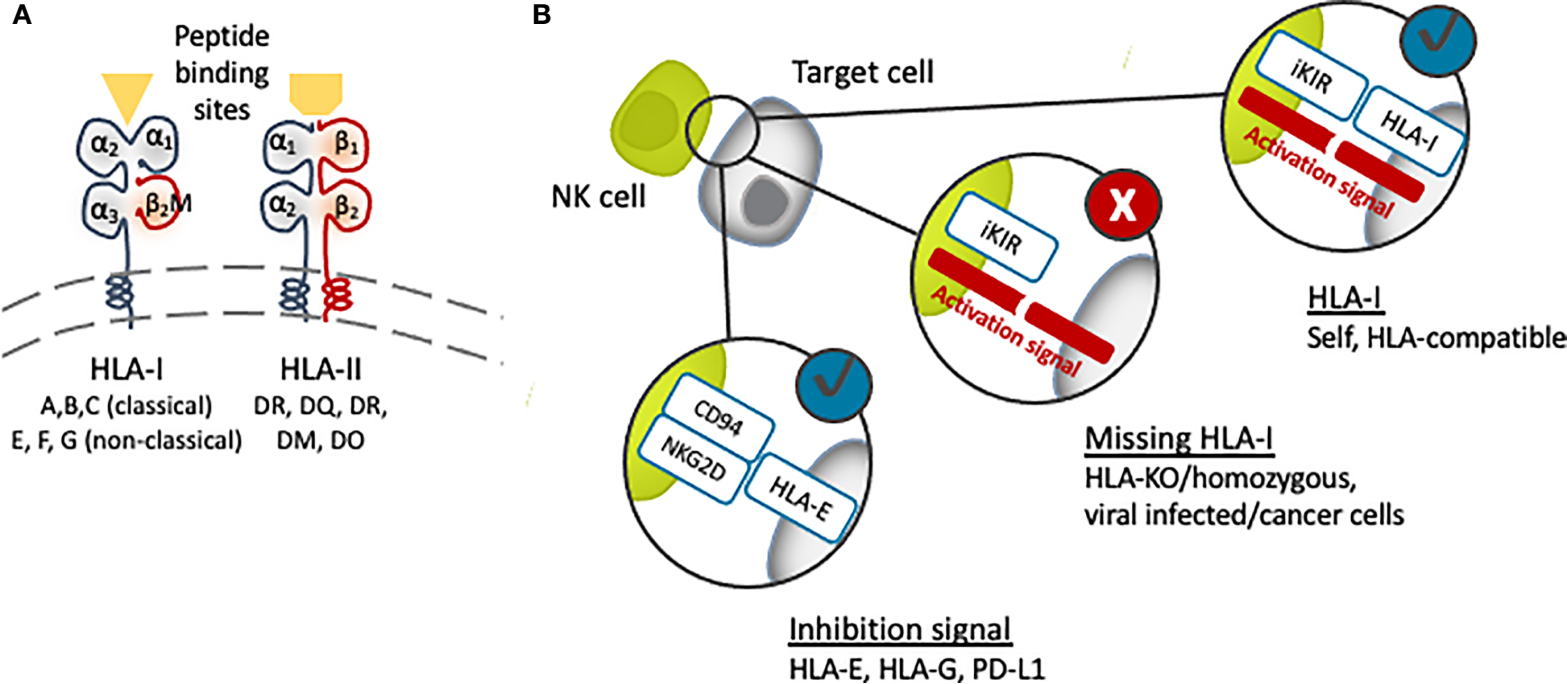
**(A)** Schematic structure of human leukocyte antigen (HLA). Targeting the β2M subunit of HLA-I will cause disruption of all HLA-I expression [major (A–C) and minor (E–G)]. **(B)** The presence of HLA-I matching with the recipient NK cells’ inhibitory KIR receptor (iKIR) will induce tolerance, while the lack or downregulation of HLA-I will trigger NK cell immunity.

Graft rejection is a major issue in allogeneic transplantations. The general mechanism of an allograft rejection is the dominant activation of the recipient’s T cells by recognizing non-self alloantigens on the donor major histocompatibility complex (MHC) antigens, which are HLAs in the case of humans. Jean Dausset described the first HLA, HLA-A2, in 1958 (41) after his observations in 1952 of donor-recipient leukocyte incompatibilities, which were then extended to tissues. Today we know that the two classes of HLA, class I, HLA-A, -B, and –C, and class II, HLA-DR, -DP, and -DQ, are encoded within chromosome 6p21 (**Figure 1**) (42). Humans carry a pair of HLA haplotypes, which are specifically distributed relative to ethnicities and geographic regions. To minimize the rejection caused by an alloimmune response, matching for the HLA haplotype between the donor and recipient is necessary, and comprehensive guidelines are applied for best practices to ensure clinical benefits to the patients (43–45).

HLA class I are cell surface proteins built up with a polymorphic heavy chain and a conserved light chain, called β-2-microglobulin (β2M), forming together a functional heterodimer (**Figure 1**). They are found in all nucleated cells and platelets, although their expression in iPSCs is lower compared to somatic cells (46). The main function of HLA class I molecules is to display peptide antigens to T cell receptors (TCRs) on CD8+ T cells. In contrast, CD4+ T cells receive antigenic cues from peptides mounted on HLA class II, which are expressed only in dendritic cells (DCs), macrophages, B cells and other antigen-presenting cells (APCs). When the HLA on transplanted cells is different from the host, T cells will initiate cytotoxic and other immune responses leading to graft destruction.

**Figure 2:**
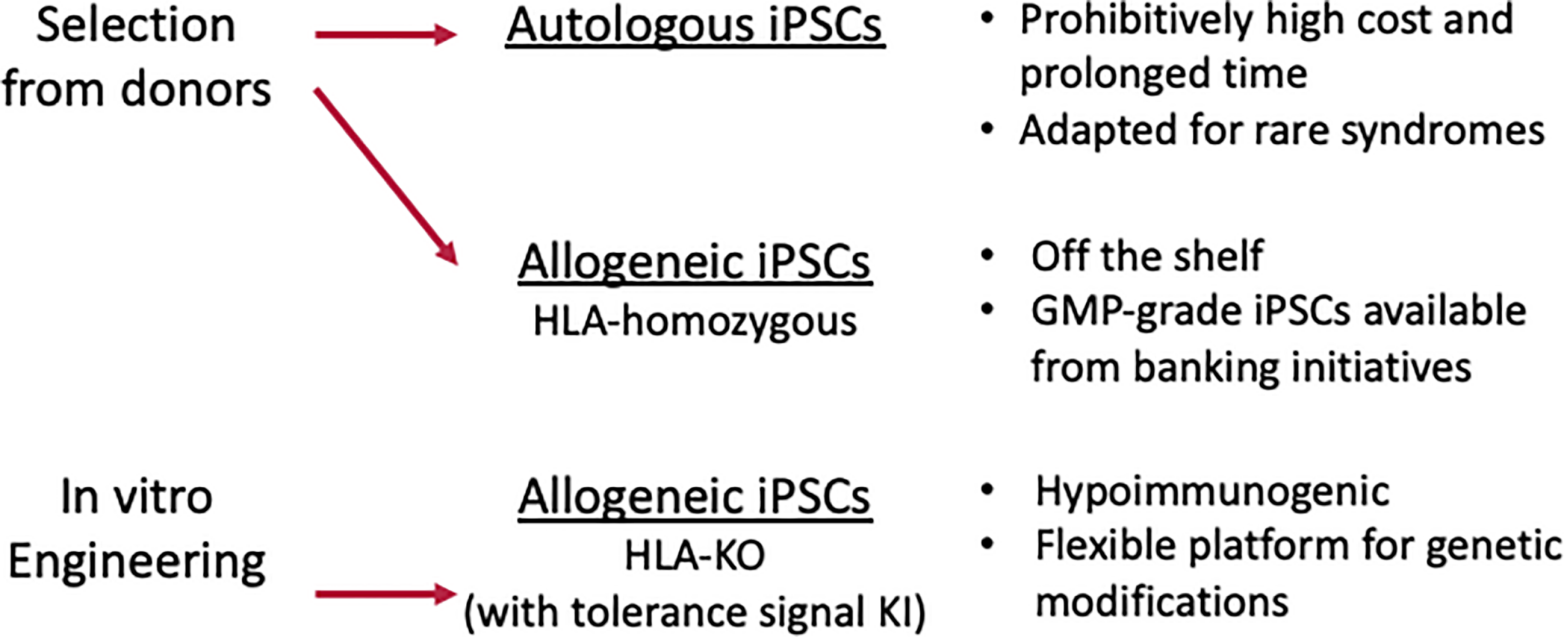
Capability of iPSCs to provide immune-compatible products.

iPSCs offer a unique opportunity to circumvent the immunological barriers to transplantation, and, as reviewed elsewhere (47, 48), a number of strategies are being explored to reduce the potential immunogenicity of the iPSC (or ESC)-derived biological transplantation material. The three main strategies, autologous, HLA homozygous and HLA depleted, are discussed here (**Figure 2**).

## Autologous iPSC-Derived Grafts

The history of allotransplantation has revealed HLA as the major driver of an immune response, but minor histocompatibility antigens and ABO antigens can be crucial alloantigens as well. Therefore, immunosuppressive agents with non-negligible side effects are usually used. However, rejection still cannot be controlled in some cases. Autologous iPSCs can be established from essentially any patient so long a small portion of skin or blood is available and provide differentiated cells that, in principle, cause no alloimmune-mediated rejection. However, there are some reports stating the immunogenicity of autologous iPSC-derived grafts, which may be due to epigenetically dysregulated expression of immunogenic proteins, somatic mutations and other alterations due to genetic instabilities and genetic manipulations (49–56).

Based on less concern of rejection and donor-derived infection, autologous iPSCs were chosen in the first clinical trial for AMD, which reported no adverse events two years after the transplantation (20). In the case of the trial for thrombocytopenia due to aplastic anemia, the person receiving iPSC-derived platelets suffers from platelet transfusion refractoriness due to an anti-platelet alloantibody, justifying the transplantation of autologous cells (35, 57).

Autologous iPSC-derived cells are often not practical, however, because as mentioned above, establishing iPSCs from patients and differentiating them to a final cell product each time requires labor- and cost-intensive logistics and the quality would vary each time (17). Indeed, not only could the therapy be prohibitively expensive, it may also not even be available in time to benefit the patient. Moreover, in the case the pathology is caused by a mutation, the generated cells will possess the same mutation if not corrected by genetic engineering.

## HLA-Homozygous iPSC Stocks as Source Cells of Allografts

As an alternative, iPSCs established from people with homozygous HLA haplotypes are available through iPSC stock projects. Currently, there is momentum to build biobanks of iPSCs with enough HLA haplotypes to supply HLA-matched cells to a wide population (8–10). iPSCs with homozygous HLA haplotypes are stocked, and good manufacturing practice (GMP) grade lines have been distributed for regenerative medicine. Homozygous haplotypes are advantageous, since they have wide compatibility potential due to the need to only match one of the two HLA haplotypes of the recipient to avoid the intense rejection mediated by T cell-HLA interactions. Thus, the mass production of uniform allogeneic off-the-shelf donor-derived material would greatly expand the number of patients who would benefit from these regenerative therapies. Accordingly, allogeneic iPSCs are predominantly preferred in clinical trials using iPSC products (**Table 1**).

However, limitations still exist, because HLA are highly polymorphic, and, as mentioned earlier, epitope heterogeneity greatly varies between geographic regions and ethnicities. Building stocks for a heterogeneous population is a greater logistic and financial challenge than for a relatively homogeneous population. For example, the required size of the Japanese HLA-homozygous iPS cell bank is relatively small compared with other regions (17). Moreover, banks must take in account alleles of rare frequencies, which adds to the number of cells to stock. Indeed, it becomes increasing difficult to cover a higher rate of the population. For Japan, the 10 most frequent HLA haplotypes can cover 50% of the population, but the 75 most frequent haplotypes are needed to reach 80%, and 140 to reach 90% (9, 17). Considering that each iPSC cell line needs to undergo careful characterization and regulatory safety evaluation, the workload, although feasible, is costly and time-consuming.

## HLA Depletion

**Figure 3:**
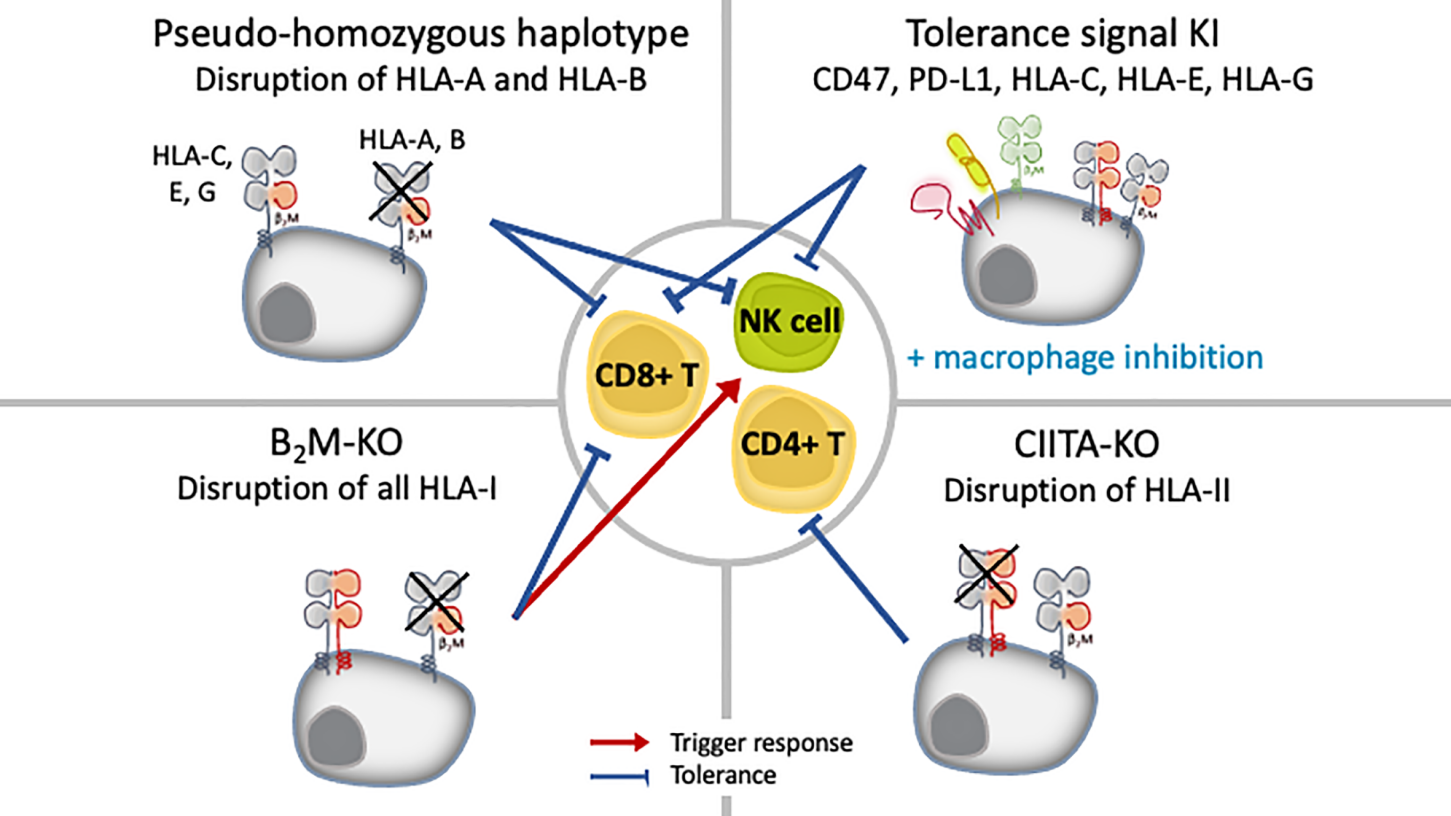
iPSC engineering strategies to inhibit allogeneic immunity and promote tolerance.

Another option is to leverage the property of iPSCs to be amenable to genetic manipulation, specifically, eliminate or downregulate the HLA expression, to avoid T cell recognition (**Figure 3**). For most cases, the reduction of HLA class I expression have been achieved by targeting the conserved β2M chain. Researchers have access to several molecular tools to disrupt β2M expression, and the choice depends on the outcome needed. Post-transcriptional manipulation by RNA interference (RNAi), either using small interfering RNA (siRNA) or short hairpin RNA (shRNA), stably downregulated or silenced the expression of HLA proteins in B-lymphocyte cell lines (58) in conjunction with human ESCs (59), iPSCs (60), primary human hepatocytes (61), endothelial cells (62, 63) and in a perfusion system at the whole organ level in rat kidney (64). Silencing with RNAi proved to be stable even after the iPSCs were differentiated to megakaryocytes and even platelets (60). TALENs (65), CRISPR/Cas9 (66–71) and vector-mediated gene targeting technology (72–74), on the other hand, are used to achieve complete depletion of HLA-I through β2M-targeting. The simultaneous knockout of HLA-A/B/C by multiplex CRISPR/Cas9 has also been reported (75).

Notably, in those studies, the complete knockout of HLA was found stable after the ESC/iPSCs were differentiated, and no off-target integration or non-specific cleavage events were observed (66, 74). However, current technology cannot rule out off-target events and potential unwanted cell behavior. Introducing modalities such as suicide genes or other safeguards to eliminate the possibility of grafts with uncontrolled growth is recommended (76). Two major suicide genes studied are thymidine kinase gene of the herpes simplex virus (HSV-TK), which can be switched on with ganciclovir treatment, and inducible caspase-9 (iCas9), which can be activated with a synthetic chemical inducer of dimerization (CID) treatment. To overcome its inactivation in proliferating cells, HSV-TK gene was transcriptionally linked to a cell-division gene CDK1 (77). Further, the combination of HSV-TK and iCas9 can kill all cell types or specifically undifferentiated ESCs/iPSCs (78). New technologies such as synthetic microRNA switches can also be applied to eliminate unwanted cells safely (79, 80).

Both knockdown and knockout have distinct advantages. Because CD8+T cell immunity can be triggered by even a very low number of HLA-I-peptide complexes (81), complete knockout of HLA class I can enable total invisibility from T cell cytotoxicity. This approach is also applicable for CD4+ T cells and HLA class II-mediated antigen presentation, for which disruption is achieved by targeting class II MHC transactivator (CIITA) genes (66–69). Additionally, neither approach seems to be detrimental to cell function after differentiation to megakaryocytes and platelets (70, 71), cardiomyocytes (67, 68) or RPE cells (66). However, because HLA class I molecules are also ligands that negatively regulate the activation of NK cells, the complete loss of HLA class I risks triggering a response by NK cells through “missing self” immunity, thus, residual expression instead of complete removal of HLA class I might be preferential to prevent graft rejection by NK cells.

## Evading NK Cell Response Against HLA-Homozygous or HLA-Depleted Cells

NK cells are the innate counterpart of cytotoxic CD8+ T cells, presenting cytotoxic activities and releasing cytokines such as interferon-γ (IFNγ) upon target cell recognition. NK cells recognize “missing self” cells that have downregulated the expression of HLA class I (11–13), such as virus-infected cells, tumor cells and experimentally HLA-mismatch allografts. The balance between activation and inhibitory signals regulates NK cell activity, and HLA class I molecules act as ligands that inhibit NK cell activation through killer immunoglobulin-like receptors (KIR) and CD94/NKG2A heterodimer (**Figure 1**). Among HLA class I molecules, all HLA-C molecules and some HLA-A and HLA-B are ligands for inhibitory KIRs. HLA-C molecules are divided into C1 and C2 groups based on the group of KIR they bind. Accordingly, one study showed that endothelial cells generated from HLA-C1/C1 iPSCs were killed by NK cells from an HLA-C1/C2 individual *in vitro*, as they were regarded as missing C2 (82). This “missing self HLA-C” immune response demonstrates the importance of considering the rejection of HLA-homozygous and HLA-depleted iPSC-derived allografts by host NK cells.

Attempts to promote tolerance against NK cells for transplanted iPSC-derived cells have come from a deeper understanding of the involvement of HLA-C, non-classical HLA class I (HLA-E, -F and -G) and self-signal (CD47 and PD-L1) pathways in cancer evasion as well as in physiological immune privileged sites, particularly the maternal-placenta interface (**Figure 3**) (83–86). In early studies, isoforms of HLA-G, a quasi-monomorphic HLA, were expressed in HLA class I-deficient or HLA class I-expressing cell lines and proved to be protective against NK cell cytotoxic activity in both cases (87, 88). *In vitro*, the forced expression of HLA-G induced an immunosuppressive phenotype in human ESCs expressing moderate levels of HLA-A, -B, -C, and low levels of HLA class II as well as in their epidermal precursor derivatives, which expressed lower levels of HLA-I overall (89). Riteau et al. showed that HLA-E was also a potent inhibitor of NK cell cytotoxicity (86). In an *in vivo* monkey model, iPSC- derived RPE cells were shown to repress NK cell cytotoxicity by a mechanism dependent on HLA-E, which was naturally expressed on these cells (90). Recently, human ESCs with knock-in of only HLA-E in conjunction with the depletion of whole HLA class I by targeting β2M did not elicit an immune response from either CD8+T cells or NK cells *in vitro* and *in vivo* (72).

Another strategy exploits immune checkpoint molecules such as PD-L1 and CD47. Both are immunomodulatory molecules involved in mechanisms of self-protection or “don’t eat me” signaling. CD47 is a ubiquitous receptor whose function is to inhibit phagocytosis (91). Its overexpression in MHC class I knockout murine iPSCs was protective in a mouse model (68). Finally, the selective targeting of classical HLA class I has been proposed as a strategy to create hypo-immune cells that are “invisible” to NK cells. Indeed, the specific targeting of HLA-A in B-lymphocyte cell lines *in vitro* and the disruption of both HLA-A and -B alleles in iPSCs *in vitro* and *in vivo* reduced lysis by T cells and NK cells (58, 69, 92).

## Modelling Human Immunity in HIS Mouse Models

**Figure 4:**
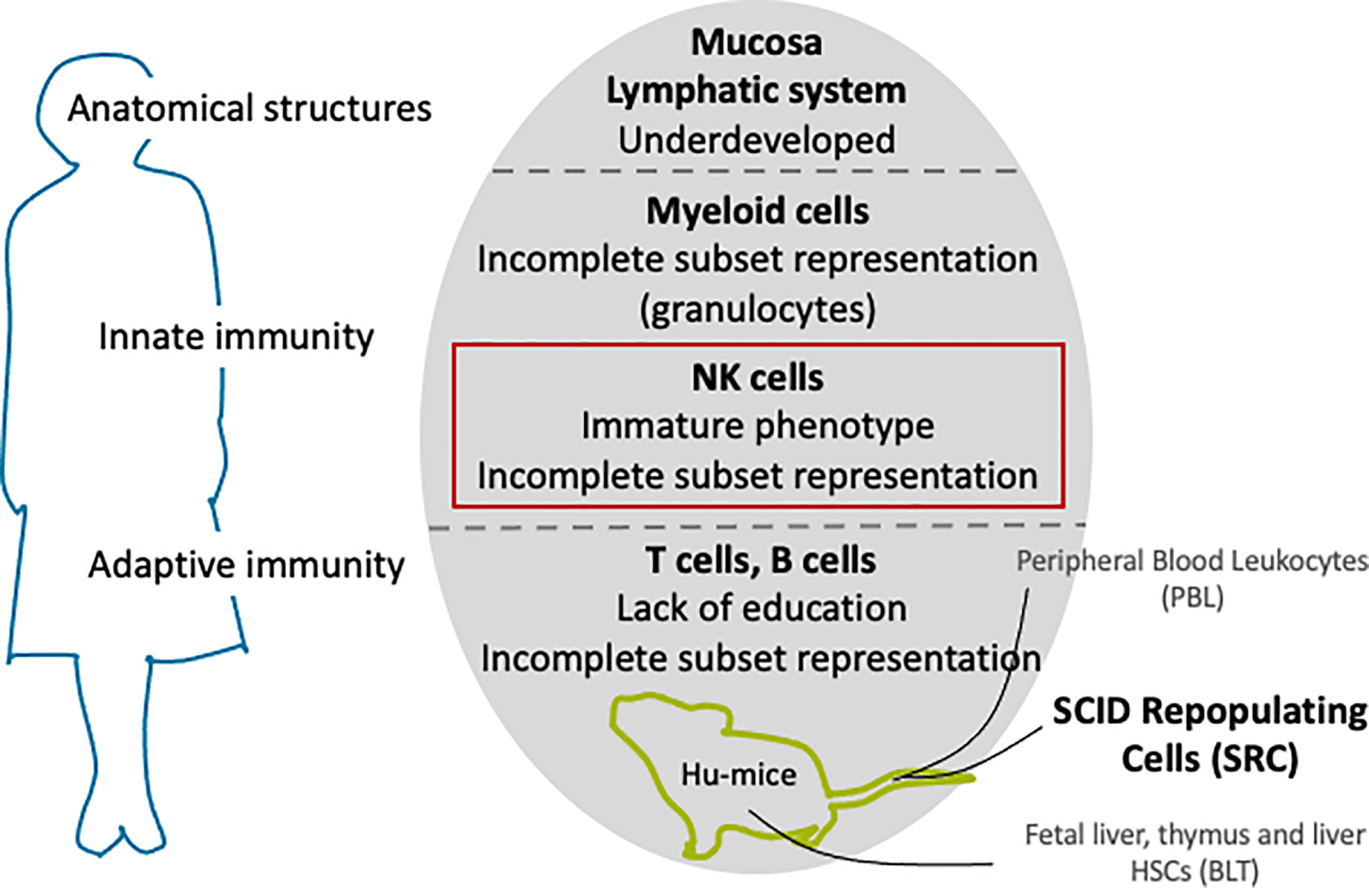
Current challenges in recounstituting a human immune system in immundeficient mice.

Transplantation tolerance and rejection mechanisms operate through several interconnected pathways involving both the adaptive and innate immune systems (93). Animal models should therefore recapitulate as many human immune system components as possible. In the human immune system, these cellular and molecular components work as an overall horizontal system that allows redundant and complementary processes to efficiently protect against internal and external threats to the body’s homeostasis. The mouse immune system mirrors the general paradigms of these complementary processes in humans, but differs in finer but significant aspects (94, 95). Although not perfect models (94, 95), mice are nonetheless instrumental pre-clinical assets for testing new therapies before moving on to human trials. In order to improve the predictive value of rodent-based studies so that mechanistic insights into immune responses are not “lost in translation” but highly relevant for transplantation studies, immune-deficient mice can be engrafted with a functional human immune system (**Figure 4**). These models will hereafter be referred to as HIS mice.

Three sources of human hemato-lymphoid cells or tissues are commonly transplanted into immunodeficient mice to stably reconstitute the human immune system. 1) In the hu-PBL model, peripheral blood leukocytes (PBL) are isolated from human blood and then transplanted into mice. 2) In the hu-SRC model, the cells transplanted are human severe combined immunodeficiency (SCID) repopulating cells (SRC), that is to say: CD34+ hematopoietic stem cells (HSCs) from umbilical cord blood (UCB), fetal liver, bone marrow (BM) or granulocyte colony-stimulating factor (G-CSF) mobilized peripheral blood. 3) In the last model, hu-BLT (BM/liver/thymus), a combination of fetal liver and thymus fragments is injected under the kidney capsule with fetal liver HSCs. Choosing one model over the other will lead to varying outcomes in the engraftment level or repopulation allotment (96). Briefly, the hu-PBL model has the advantage of grafting readily mature lymphoid cells and has a high reconstitution of CD3+ T cells, while the hu-SRC and hu-BLT models employ more primitive stem cells. As a consequence, the hu-PBL model provides a relatively easy and straightforward recapitulation of the mature immune system, but also the least physiological. Furthermore, it easily develops xeno-GVHD, which limits the experimental window period. The hu-SRC model takes a comparatively longer time to achieve reconstitution but provides the multilineage development of hematopoietic cells and can recapitulate the development of the endogenous human immune system. However, innate immune cells such as myeloid lineages and NK cells as well as T cells are less developed. In addition, xeno-GVHD could still develop, but this can be overcome by the depletion of mouse MHC molecules (97). The hu-BLT model is reputed to have the highest level of human cell reconstitution and allows the robust reconstitution of educated T cells. However, it requires high technical skills to produce as well as fetal human tissue, thus, its availability is very limited.

The development of an HIS following the xenotransplantation of human cells is not tolerated unless the mice are severely immunocompromised first. Humanization of the mouse immune system is built on three main steps of genetic manipulation and breeding. Historically, the first genetic mutant immunodeficient mice was CB17-*scid* mice discovered in 1983, in which severe combined immunodeficiency (scid) affecting T and B cells resulted from the spontaneous homozygous loss of a functional mutation in the *PRKDC* gene (protein kinase, DNA-activated, catalytic polypeptide) (98). However, the mutation did not mute the innate immune system, and murine adaptive cells progressively reconstituted as the mice aged, which resulted in the low engraftment of human cells. In a subsequent manipulation, the scid mutation was crossed in non-obese diabetic (NOD) mice (NOD-*scid*), which resulted in lower innate immune system function and resolved the diabetes from the NOD background, which was T-cell mediated (99, 100). NOD-*scid* mice have been extensively used in xenotransplantation studies, but they are impeded by a characteristically short lifespan and predisposition to lymphoma, a still relatively low level of chimerism, and the progressive “leaking” of T and B cells. Recombination-activating gene (Rag)-1 and Rag-2 knockout mice helped to reduce the reappearance of the adaptive immune system but resolved neither the tendency of the NOD-*scid* to develop lymphomas nor the engraftment issues. The last major genetic manipulation, namely the disruption of murine cytokine signaling by targeting the interleukin-2 (IL-2) receptor γ chain locus, resulted in higher chimerism levels and more silencing of both adaptive and innate cells. The resulting immunodeficient *IL2rg^null^* mice were then crossed with various stains such as NOD-*scid* to produce the NOD-*scid* gamma strain (NSG) and in the same manner the NOD-*Rag1* gamma (NRG) and BALB/c- *Rag2* gamma (BRG) strains.

## Enhancing NK Cell Reconstitution in HIS Mouse Models

NSG-, NOG-, and BRG-based HIS mice have had an important beneficial impact in biomedical research but are more inclined toward modelling T and B cells and do not adequately reconstituted innate effectors, particularly myeloid cells and NK cells. Human NK cells constitute 10% to 15% of human peripheral lymphocytes, but were found to be around 1% to 3% in HIS mice (101). Despite their poor representation, NK cells have been identified in various organs, their phenotype described, and their function (cytotoxicity and cytokine production) assessed in hu-SRC (102–105) and hu-BLT (106) models. Additionally, several approaches have been studied over the decade to successfully reconstitute more mature human NK cells with cytotoxic function *in vivo* or *ex vivo*.

## Exogenous Supply of Human Cytokines

Human cytokine supplementation has quickly proven to be useful for NK cell proliferation and differentiation in HIS mice (101, 104, 105, 107). Human cytokines can be either repeatedly administered or their expression introduced by cytokine-encoding virus vectors or plasmids. One such cytokine considered crucial for NK cell development is IL-15, which, along with IL-2 and IL-7, belongs to the family of cytokines that shares common γ chain (γc) as their receptor subunit to convey activation signals (108). The binding of IL-15 to the β and γ heterodimer complex of IL-15 receptor (IL-15Rβγ) requires trans-presentation in complex with IL-15Rα, which is expressed on myeloid-lineage cells such as monocytes and dendritic cells and also stromal cells (108). Indeed, the injection of human IL-15/IL-15Rα complex induced extensive human NK cell expansion and differentiation toward the late stage CD56^lo^CD16+ and KIR+ phenotypes in BRG mice receiving human CD34+ fetal liver cells, although the organ reconstitution was low in lymphoid organs (101). Reconstitution in mice was enhanced also by IL-15 supplementation *via* plasmids that express IL-15 and FLT3 ligand in mice (107). or by adenovirus vectors that express human IL-15 (101). The introduction of human FLT3 ligand in adenovirus vector alone can also expand human myeloid and NK cells in HIS mice (109). In this case, human FLT3 ligand primarily expands the myeloid cells, which in turn provide human IL-15/IL-15Rα to expand the human NK cells.

## Transgenic Expression of Human Cytokine Genes

The genetic engineering of interleukins in HIS mice models can increase the number of NK cells in lymphoid and non-lymphoid organs (110–112) as well as the number of CXCR6+ tissue-resident NK cells in the spleen, liver and BM (111, 112). The knock-in of human IL-15 (SRG-15 (111)) or double knock-in of IL-7 and IL-15 (hIL-7xhIL-15 KI (112)) on the other brought a significant increase in the number of human NK cells in peripheral blood compared with conventional NOG or NSG mice, achieving the physiological NK cell numbers observed in human blood in SRG-15 mice.

IL-2 and IL-7 are also two cytokines regarded fundamental to NK physiology and supplementation candidates to improve NK reconstitution. Accordingly, the transgenic expression of human IL-2 gene in a NOG mouse substrain (Hu-HSCs NOG–IL-2 Tg (110)) was able to increase the human NK cell number, but supplementation with IL-7 alone proved to be insufficient (113). The same insufficiency was found after knocking in just IL-7 in the lymphoid and non-lymphoid organs of NSG mice (112).

While researchers have increased the number of human NK cells in SRG-15 mice, the cells did not have the high degree of similarity with the NK cell repertoire of humans after stimulation (111). NK cells from SRG-15 lacked licensed (or “educated”) NK cell subsets and showed a hypo-responsiveness when challenged with K562 cells (111). The hIL-7xhIL-15 KI model also could not reflect the normal frequency of mature human NK cells, on par with hIL-15 or hIL-15-hIL/15Rα complex injection studies (112). Finally, Hu-HSCs NOG–IL-2 Tg NK cells showed a bias toward IL-2 chronic activation phenotypes, and their receptor expression did not always correspond to their natural human NK counterparts, although the KIR diversity range did (110).

## Next Generation of HIS Mice

Constitutive exposure to specific sets of human interleukins through genetic engineering seems to improve the number of NK cells in HIS mice and achieves a high reconstitution of NK cells that is superior to simple injections. However, intravenous and hydrodynamic injections of the expression plasmids as well as transgenic modifications induce systemic supra-physiological levels of human cytokines, which do not recapitulate the typical spatial and temporal expression in humans. While the replacement of mouse genes with their human counterparts allows for physiological regulation to take place, the replacement of interleukins involved in NK cell development has proven to be insufficient for mirroring human NK immunity (111, 112).

Accordingly, there seems to be more biological layers that still need to be introduced in HIS mice in order to improve the precision of human NK cell immunity reconstitution. One such path is the reconstitution of the immune cell niche in the mouse microenvironment, since NK cells typically mature through exposition to human myeloid - such as monocytes macrophages and dendritic cells- and epithelial/stromal cells, which are likely to be sources of IL-15/IL-15Rα (114). However, the mouse microenvironment-human cells cross-reactivity is limited (94, 101). Moreover, improving myeloid cell lineage representation might improve cell engraftment, as observed after the co-injection of T cell-depleted “support” cells (115). An exception is the maintenance of *ex vivo* expanded UCB-derived NK cells in HIS mice, in which autologous UCB was used for the humanized cell source (116).

Several strategies that use gene knock-in and knock-out to produce the human microenvironment and signal networks have shown optimized myelopoiesis in HIS mice: NSG-SGM3 mice, also designated NSGS, express the human cytokines stem cell factor (SCF), granulocyte-macrophage colony-stimulating factor (GM-CSF), and IL-3 (117, 118); NSGW41 mice show an inactivated mutant mouse Kit gene (119, 120); NOG-EXL mice expresses human IL-3 and GM-CSF (121); and MISTRG mice express human macrophage colony-stimulating factor (M-CSF), IL-3, GM-CSF, and thrombopoietin (TPO) (114). In the NSGS, NSGW41 and NOG-EX models, more comprehensive NK cell development is required. However, when engrafted with UCB CD34+ cells, NSGS recently showed better engraftment of CD3-CD56+ NK cells in the BM and spleen compared to NSG mice (122, 123). In contrast, abundant human myeloid cells and monocytes were found to be source of IL-15 and IL-15Rα in MISTRG mice, and a significant increase in NK cell numbers was observed in various organs of this model with effectual cytotoxic and IFNγ production (114).

While these models provide a new ground to explore NK reconstitution, no model has achieved all-inclusive human immunity. Therefore, each model should be carefully considered depending on its specific advantages and shortcomings to fit the intended research purposes and further adjustments are required. For example, NK cells need education through inhibitory receptors and, notably, HLA-I molecules, to acquire their effector function. Since mice express their own set of MHC-I, it is likely that the reconstituted NK cells are less responsive than they should be. Supporting human NK cell reconstitution in mice could be achieved with more genetic modifications, such as mouse strains modified to express human MHC-I, which have already been developed (124), and exogenously supplying NK-specific cytokines, as shown recently using injections of IL15/IL-15Rα in MSTRG mice (70). However, because immunity is an intricate and refined system, tentative tampering often comes with a price to the mice’s health. Cytokines are known to have systemic toxicity, and recent mouse strains develop life-shortening syndromes (118, 125, 126). This effect, coupled with the development of xeno-GVHD, potentially worsens in the case of IL-15 injection, since T cells also benefit from IL-15 supplementation, limiting long-term graft studies. However, as mentioned above, this may be overcome by depleting mouse MHC molecules (97).

## Evaluation of HLA-Deficient Cells in HIS Mice With NK Cells

The evaluation in HIS mice of HLA-homozygous or HLA-engineered iPSC-derived cells for regenerative therapy should be done in the presence of human NK cells in order to model the full range of NK functions. In particular, whether “missing self” grafts are rejected *in vivo* by human NK cells reconstituted in HIS mice models needs to be examined. In these experiments, components of the graft survival and tolerance response are comparatively evaluated against rejection controls, and evidences of rejection capability is essential to validate the response of the host mouse. An adequate rejection control will depend on the work outline, such the tolerance-induced condition vs. non-induced condition, autologous vs. allogeneic, wild-type vs. HLA engineered cells. It can also depend on the intrinsic properties of the graft. In the case of blood cell transfusions, the control and target populations can be mixed, administered and assessed for the ration thereafter. In the case of solid organs, two different fragments can be compared in different sites or different individuals.

The intra-graft presence of immune cells, when relevant, is the most common feature assessed in PSC-derived cell transplantation studies (72, 125–133), although its use as an estimation of actual rejection is controversial (127, 128, 133). Commonly, the assessment of immune cell infiltration is complemented by other measures of rejection, such as (but not limited to) tracking graft survival *in vivo* with fluorescence or bioluminescence imaging (BLI) (68, 69, 72, 125, 126), tissue damage analysis with staining for cell death markers (126, 127), or, when possible, by assays of the loss or gain of graft function such as blood glucose regulation by human ESC-derived islet like cells grafts (128).

As mentioned above, *in vitro* responses of NK cells from HLA-C1/C2 donors against HLA-C1/C1 homozygous iPSC-derived endothelial cells have been observed (82), raising concern of NK cells rejecting HLA-homozygous grafts. Meanwhile, an *in vivo* study that transplanted MHC homozygous monkey iPSC-derived neurons into monkey brain did not observe the infiltration of NK cells in the engrafted tissue (129) and a later preclinical study confirmed the survival of human HLA-homozygous iPSC-derived dopaminergic progenitors in mouse brain (19). In a clinical trial setting, the graft survival of HLA-homozygous RPE cells following local steroid administration was reported (21). However, *in vivo* studies are still few, and since the brain and eyes are regarded as immune privileged sites, whether HLA-homozygous cells could be rejected by NK cells should be differently assessed for other transplantation sites.

HLA-depleted or other genetically modified ESC/iPSC-derived cells have not reached clinical trials, but many have been investigated and assessed for immunogenicity against NK cells. For instance, the adoptive transfer of NK-92, an IL-2-dependant NK cell line, in immunodeficient NSG-B2m, a mouse model for beta-2 microglobulin deficiency and therefore expresses no mouse MHC class I, showed that human ESCs-derived CD45+ hematopoietic cells lacking polymorphic HLA class I was subject to NK-92-mediated lysis, but not if HLA-E was overexpressed (72). Similarly, tolerance against NK cells was reported for cells derived from HLA-A/B-knockout, HLA-C-retaining iPSCs (also preserving non-canonical HLA-E, -F, and -G, if expressed at all), or for human ESCs with HLA-ABC-KO and overexpressed HLA-G, PD-L1 and CD47 (69, 130). The studies found a functional activity by the reconstituted phenotypically human NK cells.

The rejection of HLA-depleted cells should be evaluated in SRC-transplanted HIS mice, which better recapitulate the *in vivo* response in humans. Various HLA class I-deficient cells are used to assess the immunity of HIS mice with enhanced human NKF cell development (**Table 2**). EB virus-immortalized lymphoblastoid cell lines, LCLs, and its mutant subclone deficient for endogenous HLA class I were used (105, 114). These LCLs were injected into HIS NSG mice infused with human fetal liver CD34+ cells. The rate of the HLA class I-deficient subset was smaller in the spleen, especially when the mice were pretreated with poly I:C, which had likely induced IL-15 and IL15Rα expression on myeloid cells (105). In a similar experimental design, the rate of the HLA class I-deficient subset was less in the spleen of HIS MISTRG mice compared with HIS NSG mice (114). These results indicate that human NK cells in HIS mice can specifically reject HLA class I-deficient cells if they are quantitatively and/or functionally sufficient. However, caution is required, as it later became clear that the defect of HLA expression was not confirmable in the deposited cells (134). Alternatively, cell lines that lack the expression of HLA class I, such as the leukemia-derived K562 cells, have been explored (110, 111). The growth of subcutaneously inoculated K562 cells was suppressed in HIS NOG-IL-15 Tg mice transferred with human peripheral blood NK cells compared with non-transferred mice (110). Another study co-injected K562 cells together with HLA class I-expressing Raji cells to reveal that the growth rate of K562 cells was lower in HIS SRG-15 mice than in HIS NSG or SRG mice (111). Meanwhile, one study grafted B cell acute lymphoblastic leukemia patient cells into HIS NSG mice reconstituted with parent-derived BM HSCs primed with IL-15 and poly I:C. There were fewer KIR mismatched leukemia cells in the BM of these mice than non-humanized NSG mice, indicating reconstituted NK cell-dependent tumor lysis (135).

**Table 2:** Rejection of HLA-deficient cells in HIS Hu-SRC mice *in vivo*.

Mouse strain	Human cytokine supply	HSC source	Target cells	Analysis	Reference
NSG	Indirect via poly I:C injection	UCB	LCL721.221, LCL721.45	12 h spleen FCM [vs. poly I:C(-)]	Strowig et al. (105)
MITRG /MISTRG	M-CSF/ IL-3/ GM-CSF/ TPO overexpression	UCB	LCL721.221, LCL721.45	12 h spleen FCM [vs. hu-NSG]	Rongvaux et al. (114)
NSG	IL15/IL15Rα, poly I:C injection	Parent-derived BM (KIR mismatch)	Patient-derived B-ALL cells	20 h BM	Kübler et al. (132)
NOG-IL-2-Tg	IL-2 overexpression	UCB	K562 leukemia cells	Tumor size [vs. non-Tg]	Katano et al. (110)
SRG-15	IL-15 overexpression	Fetal liver	K562 leukemia cells	24 h, FCM	Herndler-Brandstetter et al. (111)
MITRG /MISTRG	M-CSF/ IL-3/GM-CSF/ TPO overexpression + IL15/IL15Rα injection	UCB	iPSC-derived B2M-/- HPCs and platelets	6h Blood, FCM	Suzuki et al. (70)

UCB, umbilical cord blood; BM, bone marrow; HPCs, hematopoietic progenitor cells; WT, wild type; FCM, flow cytometry.

While these models successfully rejected “missing self-HLA” cells by human NK cells in HIS mice, all cells used were malignant and thus may not be applicable to regenerative medicine settings, in which non-tumor allografts would be administered. In this regard, we recently showed that HIS MSTRG mice treated with IL-15/IL-15Rα to enhance human NK cell reconstitution significantly rejected HLA class-I knockout iPSC-derived hematopoietic progenitor cells (70). Interestingly, this model also showed that HLA class I-knockout iPSC-derived platelets were not rejected by NK cells *in vivo*. These findings show the importance of assaying each cell type *in vivo* for NK cell immunogenicity.

Besides evaluating the “missing self” response, NK cells could target allogeneic and autologous contaminating undifferentiated human iPSCs that are downregulated for HLA class I (132). The proper reconstitution of NK cells is therefore indispensable for assessing the risk of teratomas formation in HIS mice. This targeting also argues for caution in the potential use of immune-suppressants in iPSC-derived regenerative therapies, since host NK cells can mitigate the development of teratomas if not managed by other strategies such as irradiation in anucleate cell products (136) and introducing suicide genes (76–78) or drug-resistant miRNA switches (79, 80).

## Conclusion

The proper modeling of NK cell immunity is fundamental to assessing the complete immunogenicity profile of new allotransplantable materials with HLA-homozygous or HLA-depleted phenotypes derived from iPSCs. In this regard, HIS mice using SRC as the source cells are a generally available system that represent a chimeric system inhabited by an endogenous human immune system. Thus, they could provide pre-clinical models to recapitulate the allograft rejection in human transplantation therapies for pre-clinical application. Since the development of HIS mice with human NK cells has only developed recently, most of these mice have been used to generally evaluate the rejection of malignant cells lacking HLA class I. However, but as the field of iPSC-based regenerative medicine progress, it is expected that their application against non-malignant iPSC-derived allografts will be extensively studied. There are still issues to overcome such as a long lifespan with stable engraftment to assess chronic allograft rejection and also reproducibility of the reconstitution, as the most common SRCs, UCB HSCs, are abundantly available without invasive procedure but will vary between donors. While further optimization is required to completely and reproducibly reconstitute the human immune system, the general availability and sophisticated engineering of HIS mice should advance experimental cell replacement therapies and regenerative medicine.

## Author Contributions

All authors wrote and reviewed the manuscript. All authors contributed to the article and approved the submitted version.

## Funding

This work was supported in part by Core Center for iPS Cell Research (JP20bm0104001, NS), Projects for Technological Development (JP20bm0404037, NS) and The Program for Technological Innovation of Regenerative Medicine (JP20bm0704051, NS) from the Japan Agency for Medical Research and Development (AMED) and by Monbukagakusho (MEXT) scholarship from the Japanese Government (CF). Laboratory of Hematopoietic Stem Cell Engineering is supported with an unrestricted grant from the Chemo-Sero-Therapeutic Research Institute (KAKETSUKEN) (TM). The funder was not involved in the study design, collection, analysis, interpretation of data, the writing of this article or the decision to submit it for publication.

## Conflict of Interest

NS has applied for patents related to this manuscript.

The remaining authors declare that the research was conducted in the absence of any commercial or financial relationships that could be construed as a potential conflict of interest.

## References

[1] Takahashi K Yamanaka S. Induction of Pluripotent Stem Cells from Mouse Embryonic and Adult Fibroblast Cultures by Defined Factors. Cell (2006) 126(4):663–76. 10.1016/j.cell.2006.07.024 16904174

[2] Takahashi K Tanabe K Ohnuki M Narita M Ichisaka T Tomoda K. Induction of Pluripotent Stem Cells from Adult Human Fibroblasts by Defined Factors. Cell (2007) 131(5):861–72. 10.1016/j.cell.2007.11.019 18035408

[3] Yu J Vodyanik MA Smuga-Otto K Antosiewicz-Bourget J Frane JL Tian S. Induced Pluripotent Stem Cell Lines Derived from Human Somatic Cells. Science (2007) 318(5858):1917–20. 10.1126/science.1151526 18029452

[4] Park I-H Zhao R West JA Yabuuchi A Huo H Ince TA. Reprogramming of human somatic cells to pluripotency with defined factors. Nature (2008) 451(7175):141–6. 10.1038/nature06534 18157115

[5] Williams LA Davis-Dusenbery BN Eggan KC. SnapShot: Directed Differentiation of Pluripotent Stem Cells. Cell (2012) 149(5):1174–1174.e1. 10.1016/j.cell.2012.05.015 22632979

[6] Blau HM Daley GQ. Stem Cells in the Treatment of Disease. N Engl J Med (2019) 380(18):1748–60. 10.1056/NEJMra1716145 31042827

[7] Yamanaka S. Pluripotent Stem Cell-Based Cell Therapy—Promise and Challenges. Cell Stem Cell (2020) 27(4):523–31. 10.1016/j.stem.2020.09.014 33007237

[8] Turner M Leslie S Martin NG Peschanski M Rao M Taylor CJ. Toward the Development of a Global Induced Pluripotent Stem Cell Library. Cell Stem Cell (2013) 13(4):382–4. 10.1016/j.stem.2013.08.003 24094319

[9] Umekage M Sato Y Takasu N. Overview: an iPS cell stock at CiRA. Inflamm Regen (2019) 39:17. 10.1186/s41232-019-0106-0 31497180 PMC6717959

[10] Sullivan S Ginty P McMahon S May M Solomon SL Kurtz A. The Global Alliance for iPSC Therapies (GAiT). Stem Cell Res (2020) 49:102036. 10.1016/j.scr.2020.102036 33212350

[11] Lanier LL. Up on the tightrope: natural killer cell activation and inhibition. Nat Immunol (2008) 9(5):495–502. 10.1038/ni1581 18425106 PMC2669298

[12] Vivier E Raulet DH Moretta A Caligiuri MA Zitvogel L Lanier LL. Innate or Adaptive Immunity? The Example of Natural Killer Cells. Science (2011) 331(6013):44–9. 10.1126/science.1198687 PMC3089969 21212348

[13] Long EO Kim HS Liu D Peterson ME Rajagopalan S. Controlling natural killer cell responses: integration of signals for activation and inhibition. Annu Rev Immunol (2013) 31:227–58. 10.1146/annurev-immunol-020711-075005 PMC3868343 23516982

[14] Allen TM Brehm MA Bridges S Ferguson S Kumar P Mirochnitchenko O. Humanized immune system mouse models: progress, challenges and opportunities. Nat Immunol (2019) 20(7):770–4. 10.1038/s41590-019-0416-z PMC7265413 31160798

[15] Zarzeczny A Scott C Hyun I Bennett J Chandler J Chargé S. iPS Cells: Mapping the Policy Issues. Cell (2009) 139(6):1032–7. 10.1016/j.cell.2009.11.039 20005794

[16] Lowenthal J Lipnick SL Rao M Hull SC. Specimen collection for induced pluripotent stem cell research: harmonizing the approach to informed consent. Stem Cells Transl Med (2012) 1(5):409–21. 10.5966/sctm.2012-0029 PMC3659701 23197820

[17] Saito MK Matsunaga A Takasu N Yamanaka S. Donor Recruitment and Eligibility Criteria for HLA-Homozygous iPS Cell Bank in Japan. In: Ilic D, editor. Stem Cell Banking. New York, NY: Springer New York: Stem Cell Biology and Regenerative Medicine (2014). p. 67–76. Available at: http://link.springer.com/10.1007/978-1-4939-0585-0_7.

[18] Trounson A McDonald C. Stem Cell Therapies in Clinical Trials: Progress and Challenges. Cell Stem Cell (2015) 17(1):11–22. 10.1016/j.stem.2015.06.007 26140604

[19] Doi D Magotani H Kikuchi T Ikeda M Hiramatsu S Yoshida K. Pre-clinical study of induced pluripotent stem cell-derived dopaminergic progenitor cells for Parkinson’s disease. Nat Commun (2020) 11(1):3369. 10.1038/s41467-020-17165-w 32632153 PMC7338530

[20] Mandai M Watanabe A Kurimoto Y Hirami Y Morinaga C Daimon T. Autologous Induced Stem-Cell–Derived Retinal Cells for Macular Degeneration. N Engl J Med (2017) 376(11):1038–46. 10.1056/NEJMoa1608368 28296613

[21] Sugita S Mandai M Hirami Y Takagi S Maeda T Fujihara M. HLA-Matched Allogeneic iPS Cells-Derived RPE Transplantation for Macular Degeneration. J Clin Med (2020) 9(7):2217. 10.3390/jcm9072217 PMC7408794 32668747

[22] Bloor AJC Patel A Griffin JE Gilleece MH Radia R Yeung DT. Production, safety and efficacy of iPSC-derived mesenchymal stromal cells in acute steroid-resistant graft versus host disease: a phase I, multicenter, open-label, dose-escalation study. Nat Med (2020) 26(11):1720–5. 10.1038/s41591-020-1050-x 32929265

[23] Pilot, Open-label, Randomised Controlled Clinical Trial to Investigate Early Efficacy of CYP-001 in Adults Admitted to Intensive Care With COVID-19. Australia: Cynata Therapeutics Limited (2020). Available at: https://clinicaltrials.gov/ct2/show/NCT04537351. Report No.: NCT04537351.

[24] A Study of transplantation of autologous induced pluripotent stem cell (iPSC) derived retinal pigment epithelium (RPE) cell sheet in subjects with exudative age related macular degeneration. Japan: RIKEN, Laboratory for Retinal Regeneration (2013). Available at: https://upload.umin.ac.jp/cgi-open-bin/ctr_e/ctr_view.cgi?recptno=R000013279. Report No.: UMIN000011929.

[25] UMIN Clinical Trials Registry. A Study of transplantation of allogenic induced pluripotent stem cell (iPSC) derived retinal pigment epithelium (RPE) cell suspension in subjects with neovascular age related macular degeneration. Japan: Kobe City Eye Hospital (2017). Available at: https://upload.umin.ac.jp/cgi-open-bin/ctr_e/ctr_view.cgi?recptno=R000029894. Report No.: UMIN000026003.

[26] Japan Registry of Clinical Trials (jRCT). Safety study using allogenic iPSC-retinal sheets for patients with retinitis pigmentosa (iRERP). Japan: Kobe City Eye Hospital (2020). Available at: https://jrct.niph.go.jp/en-latest-detail/jRCTa050200027. Report No.: jRCTa050200027.

[27] A Phase I/IIa Trial for Autologous Transplantation of Induced Pluripotent Stem Cell-Derived Retinal Pigment Epithelium for Geographic Atrophy Associated With Age-Related Macular Degeneration. United States: National Eye Institute (NEI (2020). Available at: https://clinicaltrials.gov/ct2/show/NCT04339764. Report No.: NCT04339764.

[28] Japan Registry of Clinical Trials (jRCT). Safety study of regenerative therapy with allogeneic induced pluripotent stem cell-derived cardiac spheres for severe heart failure (Regenerative cardiac spheres). Japan: Keio University School of Medicine (2020). Available at: https://jrct.niph.go.jp/en-latest-detail/jRCTa032200189. Report No.: jRCTa032200189.

[29] The Study of Human Epicardial Injection With Allogenic Induced Pluripotent Stem Cell-derived Cardiomyocytes in Ischemic Heart Failure. China, Jiangsu: Help Therapeutics (2019). Available at: https://clinicaltrials.gov/ct2/show/NCT03763136. Report No.: NCT03763136.

[30] Clinical Trial of Human (Allogeneic). iPS Cell-derived Cardiomyocytes Sheet for Ischemic. Osaka: Osaka University (2021). Available at: https://clinicaltrials.gov/ct2/show/NCT04696328. Report No.: NCT04696328.

[31] Safety and Efficacy of Induced Pluripotent Stem Cell-derived Engineered Human Myocardium as Biological Ventricular Assist Tissue in Terminal Heart Failure. Germany: University Medical Center Goettingen (2020). Available at: https://clinicaltrials.gov/ct2/show/NCT04396899. Report No.: NCT04396899.

[32] Mallapaty S. Revealed: two men in China were first to receive pioneering stem-cell treatment for heart-disease. Nature (2020) 581(7808):249–50. 10.1038/d41586-020-01285-w 32405042

[33] Kyoto Trial to Evaluate the Safety and Efficacy of iPSC-derived dopaminergic progenitors in the treatment of Parkinson"s Disease. Japan: Kyoto University Hospital (2018). Available at: https://rctportal.niph.go.jp/en/detail?trial_id=JMA-IIA00384. Report No.: JMA-IIA00384.

[34] Regenerative medicine for spinal cord injury at subacute stage using human induced pluripotent stem cell-derived neural stem/progenitor cells. Japan: Keio University School of Medicine (2020). Available at: https://jrct.niph.go.jp/en-latest-detail/jRCTa031190228. Report No.: jRCTa031190228.

[35] Clinical study of autologous transfusion of iPS cell-derived platelets for thrombocytopenia (iPLAT1). Japan: Kyoto University Hospital (2020). Available at: https://jrct.niph.go.jp/en-latest-detail/jRCTa050190117. Report No.: jRCTa050190117.

[36] An Open-Label Phase 1 Study to Investigate the Safety and Efficacy of CYP-001 for the Treatment of Adults With Steroid-Resistant Acute Graft Versus Host Disease. Australia, United Kingdom: Cynata Therapeutics Limited (2020). Available at: https://clinicaltrials.gov/ct2/show/NCT02923375. Report No.: NCT02923375.

[37] A Phase I, Open-Label, Multicenter Study of FT538 as Monotherapy in Relapsed/Refractory Acute Myelogenous Leukemia and in Combination With Monoclonal Antibodies in Relapsed/Refractory Multiple Myeloma. United States: Fate Therapeutics (2020). Available at: https://clinicaltrials.gov/ct2/show/NCT04614636. Report No.: NCT04614636.

[38] A Phase I, Open-Label, Multicenter Study of FT596 as a Monotherapy and in Combination With Rituximab or Obinutuzumab in Subjects With Relapsed/Refractory B-cell Lymphoma and Chronic Lymphocytic Leukemia [Internet]. United States: Fate Therapeutics (2020). Available at: https://clinicaltrials.gov/ct2/show/NCT04245722. Report No.: NCT04245722.

[39] A Phase I Study of FT516 as Monotherapy in Relapsed/Refractory Acute Myelogenous Leukemia and in Combination With Monoclonal Antibodies in Relapsed/Refractory B-Cell Lymphoma. United States: Fate Therapeutics (2020). Available at: https://clinicaltrials.gov/ct2/show/NCT04023071. Report No.: NCT04023071.

[40] FT500 as Monotherapy and in Combination With Immune Checkpoint Inhibitors in Subjects With Advanced Solid Tumors (Phase 1). United States: Fate Therapeutics (2020). Available at: https://clinicaltrials.gov/ct2/show/NCT03841110. Report No.: NCT03841110.

[41] Dausset J. Iso-leuco-anticorps. Acta Haematol (1958) 20(1–4):156–66. 10.1159/000205478 13582558

[42] The MHC sequencing consortium. Complete sequence and gene map of a human major histocompatibility complex. Nature (1999) 401(6756):921–3. 10.1038/44853 10553908

[43] Zachary AA Leffell MS. HLA Mismatching Strategies for Solid Organ Transplantation – A Balancing Act. Front Immunol (2016) 7:575. 10.3389/fimmu.2016.00575 28003816 PMC5141243

[44] Wiebe C Nickerson P. Strategic Use of Epitope Matching to Improve Outcomes. Transplantation (2016) 100(10):2048–52. 10.1097/TP.0000000000001284 27362311

[45] Sypek M Kausman J Holt S Hughes P. HLA Epitope Matching in Kidney Transplantation: An Overview for the General Nephrologist. Am J Kidney Dis (2018) 71(5):720–31. 10.1053/j.ajkd.2017.09.021 29246419

[46] Kim E-M Manzar G Zavazava N. Human iPS cell-derived hematopoietic progenitor cells induce T-cell anergy in in vitro-generated alloreactive CD8(+) T cells. Blood (2013) 121(26):5167–75. 10.1182/blood-2012-11-467753 PMC3695361 23687092

[47] Lanza R Russell DW Nagy A. Engineering universal cells that evade immune detection. Nat Rev Immunol (2019) 19(12):723–33. 10.1038/s41577-019-0200-1 31417198

[48] Zhao W Lei A Tian L Wang X Correia C Weiskittel T. Strategies for Genetically Engineering Hypoimmunogenic Universal Pluripotent Stem Cells. iScience (2020) 23(6):101162. 10.1016/j.isci.2020.101162 32502965 PMC7270609

[49] Zhao T Zhang Z-N Rong Z Xu Y. Immunogenicity of induced pluripotent stem cells. Nature (2011) 474(7350):212–5. 10.1038/nature10135 21572395

[50] Araki R Uda M Hoki Y Sunayama M Nakamura M Ando S. Negligible immunogenicity of terminally differentiated cells derived from induced pluripotent or embryonic stem cells. Nature (2013) 494(7435):100–4. 10.1038/nature11807 23302801

[51] de Almeida PE Meyer EH Kooreman NG Diecke S Dey D Sanchez-Freire V. Transplanted terminally differentiated induced pluripotent stem cells are accepted by immune mechanisms similar to self-tolerance. Nat Commun (2014) 5(1):1–12. 10.1038/ncomms4903 PMC4075468 24875164

[52] Zhao T Zhang Z Westenskow PD Todorova D Hu Z Lin T. Humanized Mice Reveal Differential Immunogenicity of Cells Derived from Autologous Induced Pluripotent Stem Cells. Cell Stem Cell (2015) 17(3):353–9. 10.1016/j.stem.2015.07.021 PMC9721102 26299572

[53] Todorova D Kim J Hamzeinejad S He J Xu Y. Brief Report: Immune Microenvironment Determines the Immunogenicity of Induced Pluripotent Stem Cell Derivatives. Stem Cells (2016) 34(2):510–5. 10.1002/stem.2227 26439188

[54] Liu X Li W Fu X Xu Y. The Immunogenicity and Immune Tolerance of Pluripotent Stem Cell Derivatives. Front Immunol (2017) 8:645. 10.3389/fimmu.2017.00645 28626459 PMC5454078

[55] Deuse T Hu X Agbor-Enoh S Koch M Spitzer MH Gravina A. De novo mutations in mitochondrial DNA of iPSCs produce immunogenic neoepitopes in mice and humans. Nat Biotechnol (2019) 37(10):1137–44. 10.1038/s41587-019-0227-7 31427818

[56] Wagner DL Amini L Wendering DJ Burkhardt L-M Akyüz L Reinke P. High prevalence of Streptococcus pyogenes Cas9-reactive T cells within the adult human population. Nat Med (2019) 25(2):242–8. 10.1038/s41591-018-0204-6 30374197

[57] Akabayashi A Nakazawa E Jecker NS. The world’s first clinical trial for an aplastic anemia patient with thrombocytopenia administering platelets generated from autologous iPS cells. Int J Hematol (2018) 109(2):239–40. 10.1007/s12185-018-02565-y 30535854

[58] Figueiredo C Seltsam A Blasczyk R. Class-, gene-, and group-specific HLA silencing by lentiviral shRNA delivery. J Mol Med (2005) 84(5):425–37. 10.1007/s00109-005-0024-2 16520945

[59] Deuse T Seifert M Tsao PS Hua X Velden J Eiermann T. Immunobiology of naive and genetically modified HLA-class-I-knockdown human embryonic stem cells. J Cell Sci (2011) 124(23):4127–8. 10.1242/jcs.104125 21878509

[60] Börger A-K Eicke D Wolf C Gras C Aufderbeck S Schulze K. Generation of HLA-Universal iPSC-Derived Megakaryocytes and Platelets for Survival Under Refractoriness Conditions. Mol Med (2016) 22(1):274–85. 10.2119/molmed.2015.00235 PMC5023513 27262025

[61] Figueiredo C Oldhafer F Wittauer E Carvalho-Oliveira M Akhdar A Beetz O. Silencing of HLA class I on primary human hepatocytes as a novel strategy for reduction in alloreactivity. J Cell Mol Med (2019) 23(8):5705–14. 10.1111/jcmm.14484 PMC6653539 31180181

[62] Figueiredo C Eicke D Yuzefovych Y Avsar M Hanke JS Pflaum M. Low immunogenic endothelial cells endothelialize the Left Ventricular Assist Device. Sci Rep (2019) 9(1):11318. 10.1038/s41598-019-47780-7 31383930 PMC6683293

[63] Wiegmann B Figueiredo C Gras C Pflaum M Schmeckebier S Korossis S. Prevention of rejection of allogeneic endothelial cells in a biohybrid lung by silencing HLA-class I expression. Biomaterials (2014) 35(28):8123–33. 10.1016/j.biomaterials.2014.06.007 24961166

[64] Yuzefovych Y Valdivia E Rong S Hack F Rother T Schmitz J. Genetic Engineering of the Kidney to Permanently Silence MHC Transcripts During ex vivo Organ Perfusion. Front Immunol (2020) 11:265. 10.3389/fimmu.2020.00265 32140158 PMC7042208

[65] Lu P Chen J He L Ren J Chen H Rao L. Generating Hypoimmunogenic Human Embryonic Stem Cells by the Disruption of Beta 2-Microglobulin. Stem Cell Rev Rep (2013) 9(6):806–13. 10.1007/s12015-013-9457-0 23934228

[66] Petrus-Reurer S Winblad N Kumar P Gorchs L Chrobok M Wagner AK. Generation of Retinal Pigment Epithelial Cells Derived from Human Embryonic Stem Cells Lacking Human Leukocyte Antigen Class I and II. Stem Cell Rep (2020) 14(4):648–62. 10.1016/j.stemcr.2020.02.006 PMC7160308 32197113

[67] Mattapally S Pawlik KM Fast VG Zumaquero E Lund FE Randall TD. Human Leukocyte Antigen Class I and II Knockout Human Induced Pluripotent Stem Cell–Derived Cells: Universal Donor for Cell Therapy. J Am Heart Assoc (2018) 7(23):e010239. 10.1161/JAHA.118.010239 30488760 PMC6405542

[68] Deuse T Hu X Gravina A Wang D Tediashvili G De C. Hypoimmunogenic derivatives of induced pluripotent stem cells evade immune rejection in fully immunocompetent allogeneic recipients. Nat Biotechnol (2019) 37(3):252–8. 10.1055/s-0040-1705474 PMC6419516 30778232

[69] Xu H Wang B Ono M Kagita A Fujii K Sasakawa N. Targeted Disruption of HLA Genes via CRISPR-Cas9 Generates iPSCs with Enhanced Immune Compatibility. Cell Stem Cell (2019) 24(4):566–78.e7. 10.1016/j.stem.2019.02.005 30853558

[70] Suzuki D Flahou C Yoshikawa N Stirblyte I Hayashi Y Sawaguchi A. iPSC-Derived Platelets Depleted of HLA Class I Are Inert to Anti-HLA Class I and Natural Killer Cell Immunity. Stem Cell Rep (2020) 14(1):49–59. 10.1016/j.stemcr.2019.11.011 PMC6962657 31883921

[71] Norbnop P Ingrungruanglert P Israsena N Suphapeetiporn K Shotelersuk V. Generation and characterization of HLA-universal platelets derived from induced pluripotent stem cells. Sci Rep (2020) 10(1):8472. 10.1038/s41598-020-65577-x 32439978 PMC7242456

[72] Gornalusse GG Hirata RK Funk SE Riolobos L Lopes VS Manske G. HLA-E-expressing pluripotent stem cells escape allogeneic responses and lysis by NK cells. Nat Biotechnol (2017) 35(8):765–72. 10.1038/nbt.3860 PMC5548598 28504668

[73] Riolobos L Hirata RK Turtle CJ Wang P-R Gornalusse GG Zavajlevski M. HLA Engineering of Human Pluripotent Stem Cells. Mol Ther (2013) 21(6):1232–41. 10.1038/mt.2013.59 PMC3677304 23629003

[74] Wang D Quan Y Yan Q Morales JE Wetsel RA. Targeted Disruption of the β _2_ -Microglobulin Gene Minimizes the Immunogenicity of Human Embryonic Stem Cells: Disruption of B2M Reduces Immunogenicity of hESCs. Stem Cells Transl Med (2015) 4(10):1234–45. 10.5966/sctm.2015-0049 PMC4572902 26285657

[75] Hong CH. Antigen Presentation by Individually Transferred HLA Class I Genes in HLA-A, HLA-B, HLA-C Null Human Cell Line Generated Using the Multiplex CRISPR-Cas9 System. J Immunother (2017) 40(6):201–10. 10.1097/CJI.0000000000000176 28604557

[76] Jeong HC Cho SJ Lee MO Cha HJ. Technical approaches to induce selective cell death of pluripotent stem cells. Cell Mol Life Sci (2017) 74(14):2601–11. 10.1007/s00018-017-2486-0 PMC11107638 28246701

[77] Liang Q Monetti C Shutova MV Neely EJ Hacibekiroglu S Yang H. Linking a cell-division gene and a suicide gene to define and improve cell therapy safety. Nature (2018) 563(7733):701–4. 10.1038/s41586-018-0733-7 30429614

[78] Martin RM Fowler JL Cromer MK Lesch BJ Ponce E Uchida N. Improving the safety of human pluripotent stem cell therapies using genome-edited orthogonal safeguards. Nat Commun (2020) 11(1):2713. 10.1038/s41467-020-16455-7 32483127 PMC7264334

[79] Miki K Endo K Takahashi S Funakoshi S Takei I Katayama S. Efficient detection and purification of cell populations using syn- thetic MicroRNA switches. Cell Stem Cell (2015) 16(6):699–711. 10.1016/j.stem.2015.04.005 26004781

[80] Parr CJC Katayama S Miki K Kuang Y Yoshida Y Morizane A. MicroRNA-302 switch to identify and eliminate undifferentiated human pluripotent stem cells. Sci Rep (2016) 6:32532. 10.1038/Srep32532 27608814 PMC5016789

[81] Brower RC England R Takeshita T Kozlowski S Margulies DH Berzofsky JA. Minimal requirements for peptide mediated activation of CD8+ CTL. Mol Immunol (1994) 31(16):1285–93. 10.1016/0161-5890(94)90079-5 7969189

[82] Ichise H Nagano S Maeda T Miyazaki M Miyazaki Y Kojima H. NK Cell Alloreactivity against KIR-Ligand-Mismatched HLA-Haploidentical Tissue Derived from HLA Haplotype-Homozygous iPSCs. Stem Cell Rep (2017) 9(3):853–67. 10.1016/j.stemcr.2017.07.020 PMC5599245 28867344

[83] Borrego FRR Ulbrecht M Weiss EH Coligan JE Brooks AG. Recognition of Human Histocompatibility Leukocyte Antigen (HLA)-E Complexed with HLA Class I Signal Sequence–derived Peptides by CD94/NKG2 Confers Protection from Natural Killer Cell–mediated Lysis. J Exp Med (1998) 87(5):813–8. 10.1084/jem.187.5.813 PMC2212178 9480992

[84] Braud VM Allan DSJ O’Callaghan CA Söderström K D’Andrea AD Ogg GS. HLA-E binds to natural killer cell receptors CD94/NKG2A, B and C. Nature (1998) 391(6669):795–9. 10.1038/35869 9486650

[85] Dulberger CL McMurtrey CP Hölzemer A Neu KE Liu V Steinbach AM. Human Leukocyte Antigen F Presents Peptides and Regulates Immunity through Interactions with NK Cell Receptors. Immunity (2017) 46(6):1018–29.e7. 10.1016/j.immuni.2017.06.002 28636952 PMC5523829

[86] Rouas-Freiss N Gonçalves RM Menier C Dausset JC Carosella ED. Direct evidence to support the role of HLA-G in protecting the fetus from maternal uterine natural killer cytolysis. Proc Natl Acad Sci U S A (1997) 94(21):11520–5. 10.1073/pnas.94.21.11520 PMC23525 9326642

[87] Riteau B Menier C Khalil-Daher I Martinozzi S Pla M Dausset JC. HLA-G1 co-expression boosts the HLA class I-mediated NK lysis inhibition. Int Immunol (2001) 13(2):193–201. 10.1093/intimm/13.2.193 11157852

[88] Rouas-Freiss N Marchal R Kirszenbaum M Dausset JC Carosella ED. The alpha1 domain of HLA-G1 and HLA-G2 inhibits cytotoxicity induced by natural killer cells: is HLA-G the public ligand for natural killer cell inhibitory receptors? Proc Natl Acad Sci U S A (1997) 94(10):5249–54. 10.1073/pnas.94.10.5249 PMC24664 9144223

[89] Zhao L Teklemariam T Hantash BM. Heterelogous expression of mutated HLA-G decreases immunogenicity of human embryonic stem cells and their epidermal derivatives. Stem Cell Res (2014) 13(2):342–54. 10.1016/j.scr.2014.08.004 25218797

[90] Sugita S Makabe K Iwasaki Y Fujii S Takahashi M. Natural Killer Cell Inhibition by HLA-E Molecules on Induced Pluripotent Stem Cell–Derived Retinal Pigment Epithelial Cells. Invest Ophthalmol Vis Sci (2018) 59(5):1719–31. 10.1167/iovs.17-22703 29610856

[91] Jaiswal S Jamieson CHM Pang WW Park CY Chao MP Majeti R. CD47 Is Upregulated on Circulating Hematopoietic Stem Cells and Leukemia Cells to Avoid Phagocytosis Siddhartha. Cell (2009) 138(2):271–85. 10.1016/j.cell.2009.05.046 PMC2775564 19632178

[92] Torikai H Reik A Soldner F Warren EH Yuen C Zhou Y. Toward eliminating HLA class I expression to generate universal cells from allogeneic donors. Blood (2013) 122(8):1341–9. 10.1182/blood-2013-03-478255 PMC3750336 23741009

[93] Dijke IE. Immunobiology of Transplantation. In: Michel RP GJ B, editors. Pathology of Transplantation. Cham: Springer International Publishing (2016). p. 7–51. Available at: http://link.springer.com/10.1007/978-3-319-29683-8_2.

[94] Mestas J Hughes CCW. Of Mice and Not Men: Differences between Mouse and Human Immunology. J Immunol (2004) 172(5):2731–8. 10.4049/jimmunol.172.5.2731 14978070

[95] Shay T Jojic V Zuk O Rothamel K Puyraimond-Zemmour D Feng T. Conservation and divergence in the transcriptional programs of the human and mouse immune systems. Proc Natl Acad Sci U S A (2013) 110(8):2946–51. 10.1073/pnas.1222738110 PMC3581886 23382184

[96] Walsh NC Kenney LL Jangalwe S Aryee K-E Greiner DL Brehm MA. Humanized Mouse Models of Clinical Disease. Annu Rev Pathol Mech Dis (2017) 12(1):187–215. 10.1146/annurev-pathol-052016-100332 PMC5280554 27959627

[97] Brehm MA Kenney LL Wiles MV Low BE Tisch RM Burzenski L. Lack of acute xenogeneic graft- versus-host disease, but retention of T-cell function following engraftment of human peripheral blood mononuclear cells in NSG mice deficient in MHC class I and II expression. FASEB J (2019) 33(3):3137–51. 10.1096/fj.201800636R PMC6404556 30383447

[98] Bosma GC Custer RP Bosma MJ. A severe combined immunodeficiency mutation in the mouse. Nature (1983) 301(5900):527–30. 10.1038/301527a0 6823332

[99] Shultz LD Schweitzer PA Christianson SW Gott B Schweitzer IB Tennent B. Multiple defects in innate and adaptive immunologic function in NOD/LtSz- scid mice. J Immunol (1995) 154(1):180–91. 7995938

[100] Van der Loo J. Nonobese Diabetic/Severe Combined Immunodeficiency (NOD/SCID) Mouse as a Model System to Study the Engraftment and Mobilization of Human Peripheral Blood Stem Cells. Blood (1998) 92(7):2556–70. 10.1182/blood.V92.7.2556.2556_2556_2570 9746798

[101] Huntington ND Legrand N Alves NL Jaron B Weijer K Plet A. IL-15 trans-presentation promotes human NK cell development and differentiation in vivo. J Exp Med (2009) 206(1):25–34. 10.1084/jem.20082013. 19103877 PMC2626663

[102] Hiramatsu H Nishikomori R Heike T Ito M Kobayashi K Katamura K. Complete reconstitution of human lymphocytes from cord blood CD34+ cells using the NOD/SCID/γcnull mice model. Blood (2003) 102(3):873–80. 10.1182/blood-2002-09-2755 12689924

[103] Kwant-Mitchell A Pek EA Rosenthal KL Ashkar AA. Development of Functional Human NK Cells in an Immunodeficient Mouse Model with the Ability to Provide Protection against Tumor Challenge. Sandberg JK, editor. PLoS One (2009) 4(12):e8379. 10.1371/journal.pone.0008379 20027308 PMC2793015

[104] Pek EA Chan T Reid S Ashkar AA. Characterization and IL-15 dependence of NK cells in humanized mice. Immunobiology (2011) 216(1–2):218–24. 10.1016/j.imbio.2010.04.008 20627447

[105] Strowig T Chijioke O Carrega P Arrey F Meixlsperger S Rä PC. Human NK cells of mice with reconstituted human immune system components require preactivation to acquire functional competence. Blood (2010) 116(20):4158–67. 10.1182/blood-2010-02-270678 PMC2993621 20671122

[106] Calderon VE Valbuena G Goez Y Judy BM Huante MB Sutjita P. A Humanized Mouse Model of Tuberculosis. Cardona P-J, editor. PLoS One (2013) 8(5):e63331. 10.1371/journal.pone.0063331 23691024 PMC3656943

[107] Chen Q Khoury M Chen J. Expression of human cytokines dramatically improves reconstitution of specific human-blood lineage cells in humanized mice. Proc Natl Acad Sci U S A (2009) 106(51):21783–8. 10.1073/pnas.0912274106 PMC2789167 19966223

[108] Waldmann TA. The biology of interleukin-2 and interleukin-15: implications for cancer therapy and vaccine design. Nat Rev Immunol (2006) 6(8):595–601. 10.1038/nri1901 16868550

[109] Douam F Ziegler CGK Hrebikova G Fant B Leach R Parsons L. Selective expansion of myeloid and NK cells in humanized mice yields human-like vaccine responses. Nat Commun (2018) 9(1):5031. 10.1038/s41467-018-07478-2 30487575 PMC6262001

[110] Katano I Takahashi T Ito R Kamisako T Mizusawa T Ka Y. Predominant development of mature and functional human NK cells in a novel human IL-2-producing transgenic NOG mouse. J Immunol (2015) 194(7):3513–25. 10.4049/jimmunol.1401323 25712215

[111] Herndler-Brandstetter D Shan L Yao Y Stecher C Plajer V Lietzenmayer M. Humanized mouse model supports development, function, and tissue residency of human natural killer cells. Proc Natl Acad Sci U S A (2017) 114(45):E9626–34. 10.1073/pnas.1705301114 PMC5692533 29078283

[112] Matsuda M Ono R Iyoda T Endo T Iwasaki M Tomizawa-Murasawa M. Human NK cell development in hIL-7 and hIL-15 knockin NOD/SCID/IL2rgKO mice. Life Sci Alliance (2019) 2(2):e201800195. 10.26508/lsa.201800195 30936185 PMC6445396

[113] André MC Erbacher A Gille C Schmauke V Goecke B Hohberger A. Long-term human CD34+ stem cell-engrafted nonobese diabetic/SCID/IL-2R gamma(null) mice show impaired CD8+ T cell maintenance and a functional arrest of immature NK cells. J Immunol Baltim Md 1950 (2010) 185(5):2710–20. 10.4049/jimmunol.1000583 20668220

[114] Rongvaux A Willinger T Martinek J Strowig T Gearty SV Teichmann LL. Development and function of human innate immune cells in a humanized mouse model. Nat Biotechnol (2014) 32(4):364–72. 10.1038/nbt.2858 PMC4017589 24633240

[115] Lang J Weiss NA Freed BM Torres RM Pelanda R. Generation of hematopoietic humanized mice in the newborn BALB/c-Rag2null Il2rγnull mouse model: a multivariable optimization approach. Clin Immunol (2011) 140(1):102–16. 10.1016/j.clim.2011.04.002 PMC3115423 21536497

[116] Vahedi F Nham T Poznanski SM Chew MV Shenouda MM Lee D. Ex Vivo Expanded Human NK Cells Survive and Proliferate in Humanized Mice with Autologous Human Immune Cells. Sci Rep (2017) 7(1):12083. 10.1038/s41598-017-12223-8 28935883 PMC5608690

[117] Billerbeck E Barry WT Mu K Dorner M Rice CM Ploss A. Development of human CD4+FoxP3+ regulatory T cells in human stem cell factor-, granulocyte-macrophage colony-stimulating factor-, and interleukin-3-expressing NOD-SCID IL2Rγ(null) humanized mice. Blood (2011) 117(11):3076–86. 10.1182/blood-2010-08-301507 PMC3062310 21252091

[118] Nicolini FE Cashman JD Hogge DE Humphries RK Eaves CJ. NOD/SCID mice engineered to express human IL-3, GM-CSF and Steel factor constitutively mobilize engrafted human progenitors and compromise human stem cell regeneration. Leukemia (2004) 18(2):341–7. 10.1038/sj.leu.2403222 14628073

[119] Cosgun KN Rahmig S Mende N Reinke S Hauber I Schäfer C. Kit regulates HSC engraftment across the human-mouse species barrier. Cell Stem Cell (2014) 15(2):227–38. 10.1016/j.stem.2014.06.001 25017720

[120] Rahmig S Kronstein-Wiedemann R Fohgrub J Kronstein N Nevmerzhitskaya A Bornhäuser M. Improved Human Erythropoiesis and Platelet Formation in Humanized NSGW41 Mice. Stem Cell Rep (2016) 7(4):591–601. 10.1016/j.stemcr.2016.08.005 PMC5063583 27618723

[121] Ito R Takahashi T Katano I Kawai K Kamisako T Ogura T. Establishment of a Human Allergy Model Using Human IL-3/GM-CSF–Transgenic NOG Mice. J Immunol (2013) 191(6):2890–9. 10.4049/jimmunol.1203543 23956433

[122] Beyer AI Muench MO. Comparison of Human Hematopoietic Reconstitution in Different Strains of Immunodeficient Mice. Stem Cells Dev (2017) 26(2):102–12. 10.1089/scd.2016.0083 PMC5248550 27758159

[123] Wunderlich M Chou F-S Sexton C Presicce P Chougnet CA Aliberti J. Improved multilineage human hematopoietic reconstitution and function in NSGS mice. PLoS One (2018) 13(12):e0209034. 10.1371/journal.pone.0209034 30540841 PMC6291127

[124] Shultz LD Saito Y Najima Y Tanaka S Ochi T Tomizawa M. Generation of functional human T-cell subsets with HLA-restricted immune responses in HLA class I expressing NOD/SCID/IL2rγnull humanized mice. Proc Natl Acad Sci (2010) 107(29):13022–7. 10.1073/pnas.1000475107 PMC2919921 20615947

[125] Kooreman NG de Almeida PE Stack JP Nelakanti RV Diecke S Shao N-Y. Alloimmune Responses of Humanized Mice to Human Pluripotent Stem Cell Therapeutics. Cell Rep (2017) 20(8):1978–90. 10.1016/j.celrep.2017.08.003 PMC5573767 28834758

[126] Li J Li X Liang C Ling L Chen Z Wong CK. Coreceptor blockade targeting CD4 and CD8 allows acceptance of allogeneic human pluripotent stem cell grafts in humanized mice. Biomaterials (2020) 248:120013. 10.1016/j.biomaterials.2020.120013 32278152

[127] Rong Z Wang M Hu Z Stradner M Zhu S Kong H. An Effective Approach to Prevent Immune Rejection of Human ESC-Derived Allografts. Cell Stem Cell (2014) 14(1):121–30. 10.1016/j.stem.2013.11.014 PMC4023958 24388175

[128] Szot GL Yadav M Lang J Kroon E Kerr J Kadoya K. Tolerance Induction and Reversal of Diabetes in Mice Transplanted with Human Embryonic Stem Cell-Derived Pancreatic Endoderm. Cell Stem Cell (2015) 16(2):148–57. 10.1016/j.stem.2014.12.001 25533131

[129] Morizane A Kikuchi T Hayashi T Mizuma H Takara S Doi H. MHC matching improves engraftment of iPSC-derived neurons in non-human primates. Nat Commun (2017) 8(1):385. 10.1038/s41467-017-00926-5 28855509 PMC5577234

[130] Han X Wang M Duan S Franco PJ Kenty JH-R Hedrick P. Generation of hypoimmunogenic human pluripotent stem cells. Proc Natl Acad Sci (2019) 116(21):10441–6. 10.1073/pnas.1902566116 PMC6535035 31040209

[131] Perrier-Groult E Pérès E Pasdeloup M Gazzolo L Duc Dodon M Mallein-Gerin F. Evaluation of the biocompatibility and stability of allogeneic tissue-engineered cartilage in humanized mice. PLoS One (2019) 14(5):e0217183. 10.1371/journal.pone.0217183 31107916 PMC6527235

[132] Benabdallah B Désaulniers-Langevin C Colas C Li Y Rousseau G Guimond JV. Natural Killer Cells Prevent the Formation of Teratomas Derived From Human Induced Pluripotent Stem Cells. Front Immunol (2019) 10:2580. 10.3389/fimmu.2019.02580 31787975 PMC6854018

[133] Benabdallah B Désaulniers-Langevin C Goyer M-L Colas C Maltais C Li Y. Myogenic progenitor cells derived from human induced pluripotent stem cell are immune-tolerated in humanized mice. Stem Cells Transl Med (2021) 10(2):267–77. 10.1002/sctm.19-0452 PMC7848353 32881406

[134] ATCC. LCL 721221 ATCC CRL1855-1077. Available at: https://www.atcc.org/support/faqs/11472/LCL%20721221%20ATCC%20CRL1855-1077.aspx. (Accessed March 27, 2021)

[135] Kübler A Woiterski J Witte K-E Bühring H-J Hartwig UF Ebinger M. Both mature KIR+ and immature KIR– NK cells control pediatric acute B-cell precursor leukemia in NOD.Cg-Prkdcscid IL2rgtmWjl/Sz mice. Blood (2014) 124(26):3914–23. 10.1182/blood-2014-05-572743 25359989

[136] Ito Y Nakamura S Sugimoto N Arai F Nishimura S Correspondence KE. Turbulence Activates Platelet Biogenesis to Enable Clinical Scale Ex Vivo Production Article Turbulence Activates Platelet Biogenesis to Enable Clinical Scale Ex Vivo Production. Cell (2018) 174:636–48.e18. 10.1016/j.cell.2018.06.011 30017246

